# Trends in the research and development of peptide drug conjugates: artificial intelligence aided design

**DOI:** 10.3389/fphar.2025.1553853

**Published:** 2025-02-27

**Authors:** Dong-E Zhang, Tong He, Tianyi Shi, Kun Huang, Anlin Peng

**Affiliations:** ^1^ The Third Hospital of Wuhan, Hubei University of Chinese Medicine, Wuhan, China; ^2^ School of Pharmacy, Tongji Medical College and State Key Laboratory for Diagnosis and Treatment of Severe Zoonotic Infectious Diseases, Huazhong University of Science and Technology, Wuhan, China; ^3^ Tongji-RongCheng Biomedical Center, Tongji Medical College, Huazhong University of Science and Technology, Wuhan, China; ^4^ The Third Hospital of Wuhan, Tongren Hospital of Wuhan University, Wuhan, China

**Keywords:** peptide-drug conjugates, artificial intelligence, drug discovery, drug design, drug evaluation

## Abstract

Peptide-drug conjugates (PDCs) represent an emerging class of targeted therapeutic agents that consist of small molecular drugs coupled to multifunctional peptides through cleavable or non-cleavable linkers. The principal advantage of PDCs lies in their capacity to deliver drugs to diseased tissues at increased local concentrations, thereby reducing toxicity and mitigating adverse effects by limiting damage to non-diseased tissues. Despite the increasing number of PDCs being developed for various diseases, their advancements remain relatively slow due to several development constraints, which include limited available peptides and linkers, narrow therapeutic applications, and incomplete evaluation and information platforms for PDCs. Marked by the recent Nobel Prize awarded to artificial intelligence (AI) and *de novo* protein design for “protein design and structure prediction,” AI is playing an increasingly important role in drug discovery and development. In this review, we summarize the recent developments and limitations of PDCs, highlights the potential of AI in revolutionizing the design and evaluation of PDC.

## 1 Introduction

**FIGURE 1:**
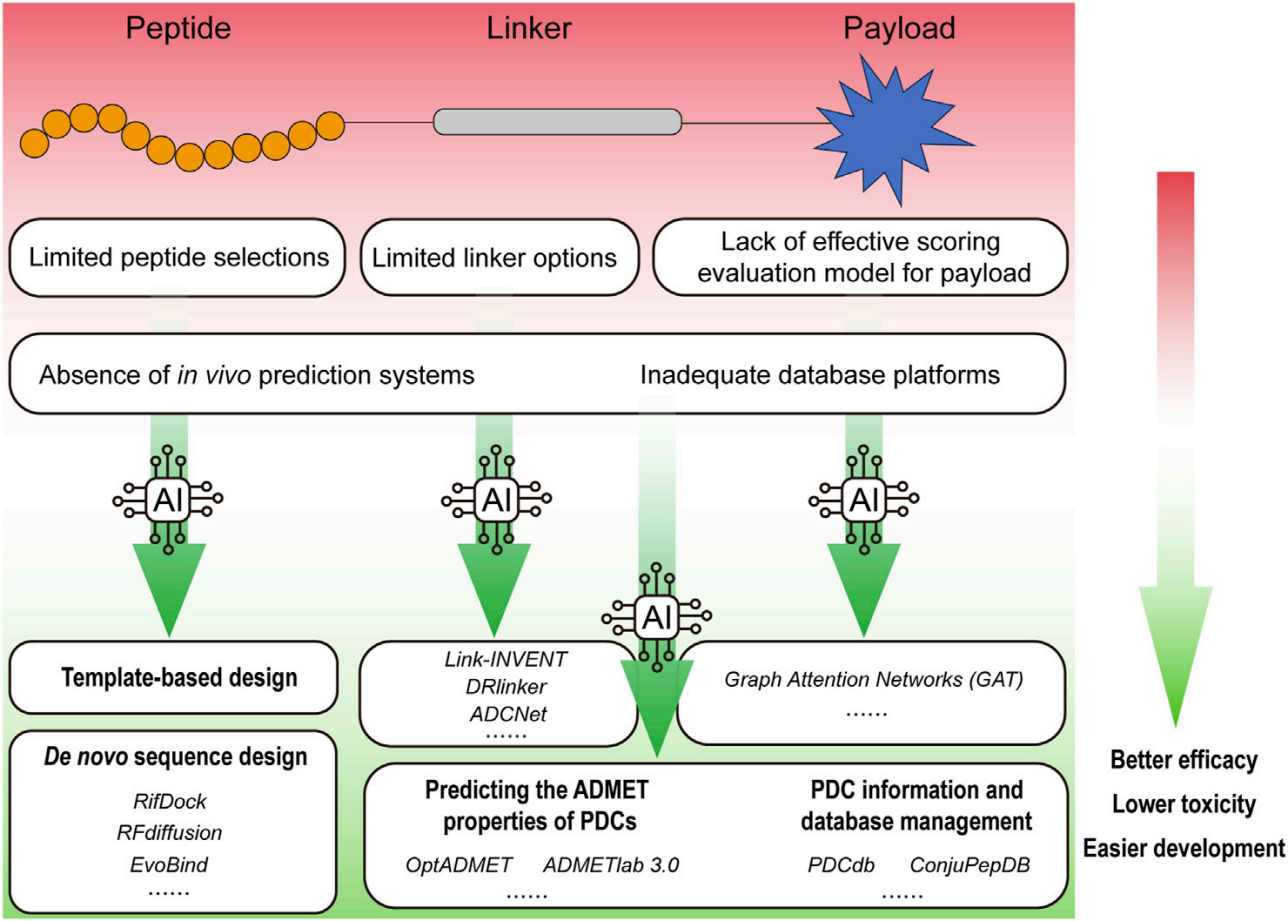
An overview of AI applications in promoting the research and development of PDCs. The factors that hinder the development of PDC drugs include limited peptide selections, limited linker options, lack of effective scoring evaluation model for payload, absence of *in vivo* prediction systems and inadequate database platforms, etc. Empowered by the AI, rationally designed PDC drugs can achieve better efficacy with lower toxicity.

Targeting therapy has emerged as a promising approach for delivering therapeutic drugs to target cells like “magic bullets” with limited damage to non-diseased tissues (Qiu et al., 2024), and has demonstrated great potentials in the treatment of cancer, chronic diseases, and infectious diseases (Srinivasarao and Low, 2017; Zhong et al., 2023). Antibody-drug conjugates (ADCs) and peptide-drug conjugates (PDCs) have been designed based on this concept with similar structure components, which comprise payloads coupled to monoclonal antibodies or multifunctional peptides through cleavable or non-cleavable linkers (Ma et al., 2017; Gong et al., 2023). Compared with ADCs, PDCs offer distinct advantages such as small molecular weight, high penetrability, low immunogenicity, high structural plasticity, and a significant reduction in adverse drug reactions (Vhora et al., 2015; Pooja et al., 2019; Gong et al., 2023; Yang Y. et al., 2023). Additionally, PDCs are associated with significantly lower production costs, making them an attractive option for developing targeted therapies. Assisted by the rapid progresses in chemical technologies, PDCs have seen fast development over the past few decades (Table 1). In 1994, The American Food and Drug Administration (FDA) approved ^111^In-DTPA-D-Phe-1-octreotide for marketing, which was the first PDC diagnostic radiology (Forssell-Aronsson et al., 2004). Following this breakthrough, a host of pharmaceutical firms and research institutions dedicate significant resources and expertise to advance the field. However, only three PDC therapeutic agents have been approved by the FDA so far, which are Lutathera at 2018 (Morgan et al., 2023), Pepaxto at 2021 (Dhillon, 2021), and Pluvicto at 2022 (Morris et al., 2024). And Pepaxto was withdrawn 7 months after its launch in the USA (Olivier and Prasad, 2022). Whereas in comparison, a total of 15 ADCs have been approved for marketing since the first ADC, Gemtuzumab Ozogamicin, received FDA approval in 2000 (Jabbour et al., 2021; Li M. et al., 2024). The cause for the obvious lag in PDCs development, include but not limited to the issues shown in Figure 1 (limited peptide selections; limited linker options; lack of effective scoring evaluation model for payload; absence of *in vivo* prediction systems; and inadequate database platforms), have restricted the development of therapeutic PDC agents.

**TABLE 1:** Current research and market approval status of representative PDC drugs.

Drug	Target	Payload	Manufacturer	Indication	Development phase	References
Lutathera	SSTR	^177^Lu	Novartis	Neuroendocrine tumors	Market	Hennrich and Kopka (2019), Kim et al. (2020)
Pluvicto	PSMA	^177^Lu	Novartis	Prostate cancer	Market	Hennrich and Eder (2022)
Pepaxto	Aminopeptidases	Melphalan	Oncopeptides	Myeloma	Market	Oriol et al. (2020), Schjesvold et al. (2020)
CBP-1018	PSMA; FLOR1	MMAE	Coherent Biopharma Suzhou Co., Ltd.	Solid tumors	Phase Ⅰ	Wu et al. (2022)
CBX-12	TOP1	Exatecan	Cybrexa Therapeutics	Solid tumors	Phase Ⅰ	Gayle et al. (2021)
TH1902	SORT1	Docetaxel	Theratechnologies	Breast cancer	Phase Ⅰ	Mäde et al. (2014), Demeule et al. (2021)
BT5528	EphA2	MMAE	Bicycle Therapeutics	Solid tumors	Phase Ⅱ	Bennett et al. (2020)
BT1718	MT1-MMP	DM1	Bicycle Therapeutics	Solid tumors	Phase Ⅱ	Gowland et al. (2021)
PEN-221	SSTR2	DM1	Tarveda Therapeutics	Neuroendocrine tumor; Small cell lung cancer	Phase Ⅱ	Whalen et al. (2019), White et al. (2019)
CBP-1008	FRα; TRPV6	MMAE	Coherent Biopharma	Solid tumors	Phase Ⅱ	Wu et al. (2022)
AEZS-108	LHRH-R	DOX	Aeterna Zentaris	Endometrial cancer	Phase Ⅲ	Worm et al. (2020)
BT8009	Nectin-4	MMAE	Bicycle Therapeutics	Urothelial cancer	Phase Ⅲ	McKean et al. (2020)
ANG1005	LRP1	PTX	Shenogen Pharma Group; AngioChem	Leptomeningeal carcinomatosis	Phase Ⅲ	Régina et al. (2008), Kumthekar et al. (2020)

Currently, the world is experiencing a new wave of technological revolution, wherein artificial intelligence (AI) has assumed a progressively significant role across an expanding array of industries. AI has achieved significant accomplishments in protein and peptide development as well as their structural validation (Qiu et al., 2024). The 2024 Nobel Prize in Chemistry was awarded for the breakthroughs in AI and *de novo* protein design, highlighting the growing importance of these technologies in drug discovery and development (Nobel Committee, 2024) (Buller et al., 2025). In the context of PDCs, AI has revolutionized traditional design paradigms. For instance, deep learning frameworks like RFdiffusion now enable *de novo* generation of cyclic cell-targeting peptides (CTPs) with 60% higher tumor affinity compared to phage-display-derived sequences (RMSD <1.5 Å) (Watson et al., 2023). Reinforcement learning platforms such as DRlinker have optimized cleavable linkers for PDCs, achieving 85% payload release specificity in tumor microenvironments versus 42% with conventional hydrazone linkers (Tan et al., 2022). Furthermore, graph neural networks (GAT) have streamlined payload screening, identifying exatecan derivatives with 7-fold enhanced bystander killing effects in multi-drug-resistant cancers (Guo et al., 2024). These AI-driven innovations address the critical limitations outlined in Figure 1, propelling PDCs from empirical design to computational-driven precision medicine. Drug development, including that of PDCs, is poised for rapid advancement with the support of AI. The PDCdb database indicates that 78% of PDCs entering clinical trials since 2022 utilized AI-optimized components, as compared to a pre-2020 < 15% rate (Sun et al., 2025). Notable examples include MP-0250, a VEGF/HGF-targeting PDC designed via AlphaFold2-guided peptide-receptor docking, which demonstrated 34% objective response in Phase II Non-Small Cell Lung Cancer (NSCLC) trials (NCT04088664), and AI-refined somatostatin analogs in Lutathera^®^ that reduced hepatotoxicity by 22% post-FDA approval (Quan et al., 2020; Morgan et al., 2023). Here, we summarize the peptides, linkers, and payloads used in PDCs; integration of AI approaches for the optimal design and prediction of PDCs are also reviewed and discussed.

## 2 Components of PDCs

Peptide-drug conjugates (PDCs) utilize peptides, linkers, and payloads to achieve targeted therapeutic effects. Traditionally, peptides in PDCs are categorized into cell-penetrating peptides (CPPs) and cell-targeting peptides (CTPs). CPPs enhance drug delivery across cell membranes via direct membrane penetration or endocytosis, with examples like HIV-TAT demonstrating transactivating activity (Derossi et al., 1994; Rizzuti et al., 2015). However, their low cellular selectivity has reduced their preference in PDC design. In contrast, CTPs, such as bombesin analogs, GnRH analogs, and RGD peptides, bind specifically to target cell receptors to facilitate internalization and lysosomal delivery (Li and Qian, 2002; Wang et al., 2021). Despite superior specificity, CTP availability remains limited, and both CPPs and CTPs suffer from enzymatic degradation, necessitating parenteral administration (Fu et al., 2023). Optimization strategies like cyclization, unnatural amino acid incorporation, and AI-driven design aim to enhance peptide stability and specificity (Wang M. et al., 2024).

Linkers in PDCs bridge peptides and payloads, balancing stability and controlled release. Cleavable linkers respond to pH (e.g., hydrazone/acetal bonds), enzymes (e.g., Val-Cit cleaved by CatB/MMPs), or redox conditions (e.g., disulfide bonds degraded by elevated glutathione in tumors) (Bargh et al., 2019; Cheng et al., 2024). PDCs with redox-sensitive linkers may only applicable to certain types of tumors, because comparison of intracellular GSH concentrations between cancer cells and normal cells indicates substantial heterogeneity across different human tumors (Gamcsik et al., 2012). Therefore, redox-sensitive PDCs may show high therapeutic potentials in breast, ovarian, head and neck, and lung cancers, which have elevated tumor GSH levels than those in disease-free tissues; whereas show limited efficacy in malignancies like brain and liver tumors in which tumor GSH concentrations fall below baseline levels observed in disease-free tissues (Gamcsik et al., 2012). Non-cleavable linkers (e.g., amide/thioether bonds) improve plasma stability but may hinder payload release, requiring optimization of length and polarity to maintain pharmacokinetic efficacy (Xu et al., 2014).

Payloads in PDCs retain activity post-conjugation and include small molecules (e.g., doxorubicin, paclitaxel) for tumor-selective cytotoxicity and radionuclides (e.g., Lutathera) for imaging/therapy (Vrettos et al., 2018; Morgan et al., 2023). Additional payloads like folic acid and cholesterol further expand functional versatility (Scomparin et al., 2015). By integrating optimized peptides, tailored linkers, and potent payloads, PDCs aim to enhance therapeutic precision and minimize off-target effects.

## 3 Key challenges in PDC development

It has been 30 years since the first PDC (^111^In-DTPA-D-Phe-1-octreotide) was approved by the FDA for marketing (Forssell-Aronsson et al., 2004). To date, about 300 PDCs have entered clinical trials, yet only three PDC therapeutics have received FDA approval for commercialization (Olivier and Prasad, 2022). Major factors that hinder the development of PDC drugs are as follows.

### 3.1 Limited peptide selections

Since the early 1920s, when insulin, composed of 51 amino acids, was isolated and commercialized, the industry of peptide drugs has seen profound development (Bliss, 1982). Thousands of peptides have been discovered, often originating from plants, animals, microorganisms, and other biological sources, and they possess many significant biological functions. While recent technological advances in proteomics, solid-phase peptide synthesis (SPPS), DNA-encoded chemical libraries (DELs), mRNA display, and phage display have accelerated novel peptide discovery, as demonstrated in recent reports (Gong et al., 2023; Collie et al., 2024; Jahandar-Lashaki et al., 2024; Kempson et al., 2024; Villequey et al., 2024), such approaches remain resource-intensive requiring significant time and financial investments. Besides, the discovery of effective druggable peptides, particularly peptidomimetics (linear/cyclic) with optimized pharmacological properties, remains critical aims to overcome common limitations such as short circulating half-lives, rapid renal clearance, and poor targeting observed in most of reported peptides (Anand et al., 2023). These characteristics present significant challenges in the development of PDCs with enhanced targeting capabilities and therapeutic efficacy (Zhang et al., 2024). According to statistics from PDCdb, currently only over a thousand peptides (including pseudopeptides and cyclic peptides) are used in PDC drugs (Sun et al., 2025).

### 3.2 Limited linker options

In PDCs, the linker serves as a pivotal bridge but is also associated with several limitations, such as inadequate plasma stability, non-specific payload release, and diminished drug efficacy post-release. Therefore, how to find linkers with reasonable therapeutic index is urgent. These issues complicate the optimization of PDC stability and cleavage, which are critical for ensuring their therapeutic efficacy and safety (Giese et al., 2021). The functional groups present in amino acids impose limitations on the diversity of feasible chemical reactions. While the selection of linkers for PDCs offers greater flexibility compared to ADCs, the PDCdb indicates that only 140 distinct linkers are currently employed in the formulation of PDC drugs (Sun et al., 2025).

### 3.3 Lack of effective scoring evaluation model for payload

The effectiveness of PDCs relies on identifying and developing payloads with a reasonable therapeutic index. Traditional methods for payload selection are limited due to their empirical nature and lack of predictive power, which limit rationale selection of payloads and PDC design (Sagar et al., 2025).

### 3.4 Absence of *in vivo* prediction systems

While *in vivo* ADMET prediction systems remain underdeveloped across drug discovery (including for small molecules), the distinct mechanisms of PDCs such as peptide-receptor binding dynamics, linker stability in systemic circulation, and payload release kinetics, create unique challenges that remain poorly addressed by existing models. Presently, there is no *in vivo* ADMET prediction system designed/available to evaluate characteristics of PDCs, including physicochemical properties, pharmacokinetic, pharmacodynamic, toxicity, etc. These characteristics are crucial for the rational design and optimization of PDCs (Yang Y. et al., 2023). For example, conventional tools optimized for small molecules (e.g., ADMETlab 3.0) may predict the behavior of PDCs due to their hybrid macromolecular architecture and context-dependent cleavage mechanisms (Yang Y. et al., 2023; Fu et al., 2024). This gap underscores the urgent need for specialized predictive frameworks tailored to PDC pharmacology.

### 3.5 Inadequate database platforms

Efficient design of PDCs require a robust prediction/evaluation system; however, current databases offer incomplete and infrequently updated information, which hamper researchers’ ability to obtain actionable insights for the design of PDCs (Deutsch et al., 2008; Wen et al., 2019).

## 4 Application of artificial intelligence in the research and development of PDCs

The 2024 Nobel Prizes in Chemistry and Physics are awarded to AI related discoveries, which recognized AI as an essential tool for the rapid advancement of scientific research today. In the field of drug discovery, AI holds great advantage in the design and discovery of new drugs, and notable successes have been achieved in the development of PDC drugs using AI.

### 4.1 AI-assisted peptide selection and design

Emerging AI applications in peptide research, such as AI-driven screening, *de novo* sequence generation, and optimization pipelines, have greatly reduced both timelines and resource expenditure compared to traditional screening methodologies (Wang C. et al., 2019; Hashemi et al., 2024). Its notable advantages enable AI-based platforms to rapidly design large number of peptides based on the properties of provided protein/peptide targets, including length, charge, binding energy (Parvatikar et al., 2023). Here, we categorize AI-based peptide design approaches into three types: template-based design, *de novo* sequence design and peptide optimization (Chen et al., 2017; Wang et al., 2020). A summary of their respective advantages and disadvantages are provided below.

Generally, many proteins have physiological or pathological binding partners, exploring this property, template-based design derives high affinity binding peptides based on protein-protein interactions (PPIs) binding motifs obtained from these known binding partners. As a groundbreaking work, AlphaFold (AF) (https://golgi.sandbox.google.com/) is capable of predicting monomer structures as well as forecasting interactions between peptides and protein receptors, particularly in the contexts where peptide-binding motifs are present. Tomer et al. offers an exhaustive comparison of the performance of AlphaFold2 (AF2) against the advanced peptide docking protocol PIPER-FlexPepDock, underscoring AF2’s potential to yield structural insights into a broad spectrum of peptide-protein complexes; moreover, they also delve into AF2’s capacity to discern binding motifs and interface hotspots, along with its efficacy in modeling interactions characterized by substantial conformational alterations in the receptor. This approach simplifies the procurement of template peptides, eliminating the necessity for intricate structural analyses, which is of paramount importance for the development of targeted peptides leveraging deep learning and AI technologies (Tsaban et al., 2022). In another example, we have recently used deep neural networks including AlphaFold2 and RoseTTAFold to model the interaction between kidney injury molecule 1 (KIM1, also known as HAVCR1) and death receptor 5 (DR5), top-scored antagonistic peptides derived from human KIM1/DR5 based on predicted binding sites significantly blocked KIM1-DR5 interaction and exhibited reno-protection effects against acute kidney injury *in vitro* and *in vivo* (Yang et al., 2021; Yang C. et al., 2023). Similarly, we managed to inhibit the liquid-solid phase separation of α-synuclein by AI-assisted rationally designing decapeptides that may attenuate the progress of Parkinson’s disease (Li X. et al., 2024; Yu et al., 2025). Although template-based design has shown principal advantages as the robustness and comparatively high success rate; on the other hand, its applications are also constrained in principle to the design of interactions with existing binding interfaces and binding partners, which precludes the exploration of novel binding sites/surfaces and sequences.

*De novo* peptide design relies solely on the sequence and/or structural information of target receptors to generate novel binding peptides, based on working mechanisms, this approach can be broadly categorized into three types.

(1) Docking optimization docks hundreds of protein scaffolds against the target receptor, with the objective of identifying conformations that exhibit favorable shape complementarity. Once suitable docking conformations are identified, optimization is initiated to achieve the lowest energy state, thereby enhancing the binding affinity between the proteins. RifDock (https://github.com/rifdock/rifdock.) serves as a paradigmatic example, which is marked by the identification of known PPIs within these structures and the subsequent development and targeting of a new set of scaffolds that incorporate these motifs against the relevant targets (Cao et al., 2022).
(2) Hotspots generation. Hotspots are critical residues that substantially contribute to the binding affinity of PPIs. By inputting a set of hotspot residues on the interaction interface, AI models autonomously generate sequences that correspond to this epitope binding, thereby enhancing the efficiency and success rate of the design. A prime example of this approach is RFdiffusion (https://github.com/RosettaCommons/RFdiffusion#binder-design), which utilizes hotspot residues on the target receptor as conditional inputs for the generation of peptide scaffolds (Watson et al., 2023).
(3) Search template. EvoBind (https://gitlab.com/patrickbryant1/binder_design) exemplifies this approach by utilizing Foldseek, a tool for protein structure alignment, to identify proteins with analogous structures and employing inverse folding algorithms for sequence design (Bryant and Elofsson, 2023). Moreover, additional approaches have been developed that expand upon methods mentioned above by utilizing protein language models to enhance the optimization of generated sequences, thereby improving the accuracy and success rates.

The application of AI in peptide engineering has demonstrated remarkable potentials in pioneering novel peptide designs and refining existing molecular structures, which may revolutionize high-quality peptide development through enhanced generation and optimization processes and has received great attention. Recent advancements include an innovative graph attention mechanism integrated with reinforcement learning (https://github.com/p1acemker/MomdTDSRL.git), which enables customized generation of target-specific peptide variants while simultaneously improving target binding affinity (Wang Q. et al., 2024). Another study develops EvoGradient (https://github.com/MicroResearchLab/AMP-potency-prediction-EvoGradient), an interpretable deep learning framework that combines antimicrobial peptide (AMP) potency prediction with AI-driven sequence modification (Wang et al., 2025), this technology facilitates automated AMP optimization through virtual screening and sequence refinement, demonstrating promise in addressing antimicrobial resistance challenges. These studies exemplify how machine learning approaches are redefining computational peptide optimization.

### 4.2 AI in linker optimization

Linker is essential for fragment-based drug discovery (FBDD), with a goal to connect two molecular fragments in a way that enhances the overall binding affinity and pharmacological property of the resulting compound. Previously, computational tools for linker design mainly rely on database searches, inherently limiting the generalizability of proposed linkers (Thompson et al., 2008). Recent breakthroughs in PROTAC, ADC, and PDC therapeutic development have been driven by AI-powered methodologies that harness deep learning and reinforcement learning to navigate the intricate challenges of linker design, marking a transformative shift in precision drug engineering.

Link-INVENT (https://github.com/MolecularAI/Reinvent) is an extension of the *de novo* molecular design platform REINVENT (Guo et al., 2023), which employs reinforcement learning to generate favorable linkers that connect molecular subunits while meeting a diverse set of objectives. Based on this platform, the trained recurrent neural network (RNN)-based generative model can be utilized to input two molecular subunits and subsequently propose ideal linkers. This platform is particularly adept at tasks such as fragment linking, scaffold hopping, and PROTAC design, which are critical for drug discovery. Link-INVENT learns to generate linkers that not only meet the physicochemical property requirements, but also exhibit desired structural features, as facilitated by a flexible scoring function that can be tailored to various multi-parameter optimization objectives. For example, Link-INVENT model demonstrates significant potential for rationally designing PROTACs capable of achieving selective dual degradation of anti-apoptotic proteins Mcl-1 and Bcl-2 (Wang Z. et al., 2019). Through the manipulation of linker length, linearity or flexibility by the Link-INVENT model, the extensive user control of Link-INVENT over the linker properties has been demonstrated.

DRlinker (https://github.com/biomed-AI/DRlinker) is another method that introduces a novel framework that harnesses deep reinforcement learning for controlling fragment linking toward compounds with specified attributes (Tan et al., 2022). This approach has demonstrated effectiveness in controlling linker length and log P, optimizing predicted bioactivity, and tackling various multi-objective tasks. Notably, DRlinker successfully generated a high percentage of compounds complying with the desired linker length and log P, and improved the pChEMBL value in bioactivity optimization. Furthermore, application of DRlinker in a quasi-scaffold-hopping study revealed its capability to generate molecules with high 3D similarity but low 2D similarity to the lead inhibitor, underscoring its potential in actual fragment-based drug design. For example, Tan et al. employed DRlinker to generate linkers for optimization of potent inhibitors targeting pantothenate synthase (Pts) from *Mycobacterium tuberculosis* (Hung et al., 2009; Tan et al., 2022). By docking top candidates, the results showed better energy scores comparing with the lead compound, which demonstrated the potential of DRlinker in FBDD.

Recent advancements demonstrate the successful application of AI-driven deep learning in optimizing linkers for ADCs. The novel ADCNet (https://github.com/idruglab/ADCNet) model integrates ESM-2 and FG-BERT multimodal language models to comprehensively analyze structure-function relationships across ADC components, including antibodies, linkers, and payloads. This approach enables high-precision prediction of critical linker properties such as stability and drug release efficiency. Trained on a rigorously curated benchmark dataset, ADCNet achieved good performance (five-fold cross-validation AUC = 0.92), with ablation studies confirming that linker feature embedding alone contributed to a 38% improvement in prediction accuracy. For the Phase III anti-TROP2 ADC SKB264, ADCNet screened 1,200 linker candidates *in silico*, prioritizing a polyethylene glycol (PEG)-based linker with pH/enzyme dual responsiveness. Experimental validation showed that AI-designed linker extended plasma half-life by 2.3-fold compared to traditional disulfide linkers, while increasing tumor-to-plasma payload release selectivity from 5:1 to 18:1 in xenograft models. This linker is now a key to improved therapeutic index of SKB264 (NCT04152499) (Hu et al., 2024). The accompanying DeepADC (https://adcnet.idruglab.cn/) platform now supports rational linker design by quantifying over 20 key parameters, including conjugation site compatibility and chemical stability, which significantly accelerate novel linker development. This progress marks the transition of AI-driven linker optimization from theoretical exploration to practical implementation, providing a robust framework for designing next-generation ADCs with enhanced efficacy and reduced toxicity (Chen et al., 2024).

The platforms mentioned above represent significant strides in the application of AI for linker optimization. These ADC successfully provide a roadmap for PDC linker optimization. For instance, DRlinker-generated thiourea linkers (Tan et al., 2022), initially validated in ADC protease stability assays, have been adapted for PDCs targeting fibroblast activation protein (FAP)-expressing tumors. In preclinical models, these linkers reduced hepatic clearance of PDCs by 40% while maintaining >80% payload release in FAP + microenvironments (Gong et al., 2023). The ability of AI to process structural characteristics of linkers in PROTACs, ADCs, and PDCs enables systematic optimization of these critical components in targeted therapies, enhancing pharmacological profiles and drug-like properties through intelligent design refinement. Furthermore, it has furnished researchers with a potent tool for identifying novel drug candidates. AI, through the integration of deep learning and reinforcement learning techniques, is capable of managing the intricacies associated with linker design, thereby heralding a transformative advancement in drug discovery research.

### 4.3 AI in payload identification and development

The efficacy of PDC payloads screening is constrained by traditional empirical methods. However, the incorporation of AI, notably Graph Attention Networks (GAT), marks a significant advancement in the rational design and discovery of PDC payloads. Although GAT has not been extensively applied to PDCs, its efficacy has been confirmed in the context of ADC payload screening.

Recent advances in the GAT have enabled the development of a quantitative bystander-killing scoring model for systematic screening and evaluation of ADC payloads. This methodology leverages the DeepChem and PyTorch Geometric frameworks, employing GAT layers to forecast the permeability of compounds. The predictive accuracy of this model was substantiated using an external dataset comprising over 80 ADC payloads in clinical use or under development, sourced from ADCdb, an ADC database (Shen et al., 2024); and this GAT-driven scoring model has been applied to identify a range of exatecan derivatives and find T-VEd9 as the optimal conjugate (Guo et al., 2024). These validations underscore the robustness of this model and its broad applicability to real-world settings, as well as the transformative impact of AI in the identification and development of ADC payloads. The GAT-based scoring model offers a novel framework for propelling ADC therapy towards clinical applications, underscoring a pivotal role of AI in tackling complex biological challenges in drug development. Given the analogous compositional structure of PDCs and ADCs, there is optimism that the GAT-based scoring model could be readily adapted for PDC payload screening.

### 4.4 AI in predictive modeling for PDCs

The *in vivo* properties are key to PDC drug design, predicting the absorption, distribution, metabolism, excretion, and toxicity (ADMET) properties of PDCs *in vivo* through AI provides an effective optimization strategy that improves success rate. Two pioneering platforms demonstrating these advancements are OptADMET and ADMETlab 3.0, which employ advanced predictive models to systematically improve the assessment of ADMET characteristics in small molecule therapeutics. These platforms not only improve the accuracy of predictions but also provide useful tools for drug design by offering in-depth analysis of key characteristics such as drug distribution, plasma protein binding, and blood-brain barrier penetration.

OptADMET (https://cadd.nscc-tj.cn/deploy/optadmet/) is an integrated web-based tool that employs data-driven chemical transformation rules for optimizing 32 different ADMET properties (Yi et al., 2024). This platform stands out due to its multi-property transformation rule database, which comprises 41,779 validated transformation rules derived from the analysis of 177,191 reliable experimental datasets. OptADMET facilitates the prediction of desirable substructure transformations, enabling researchers to balance multiple ADMET properties while deciding on the next compounds to synthesize. The strength of this platform lies in its foundation on matched molecular pairs analysis, which provides a practical approach to lead optimization by leveraging synthetic chemistry knowledge.

On the other hand, ADMETlab 3.0 (https://admetlab3.scbdd.com) is an updated comprehensive online ADMET prediction platform that boasts broader coverage, improved performance, application programming interface (API) functionality, and decision support (Fu et al., 2024). The present 3.0 version includes 119 features, an increase of 31 features compared to its predecessor, and the number of entries has increased by 50% to exceed 400,000 entries. ADMETlab 3.0 utilizes a multi-task deep message passing neural network (DMPNN) architecture, which ensures high calculation speed and superior performance in terms of accuracy and robustness. The introduction of an API in ADMETlab 3.0 caters to the growing demand for programmatic access to large amounts of data, streamlining the process of batch evaluation. Furthermore, the platform includes uncertainty estimates in prediction results, which is crucial for the confident selection of candidate compounds for further studies and experiments.

OptADMET and ADMETlab exemplify transformative progress in AI-powered predictive modeling for drug discovery. By leveraging SMILES (Simplified Molecular Input Line Entry System) notation to represent peptide-drug conjugate (PDC) molecular architectures, these platforms enable comprehensive prediction of ADMET profiles for PDC candidates, thereby streamlining drug development pipelines while mitigating financial and clinical risks. These platforms underscore the potential of AI to transform the pharmaceutical industry by providing tools to make informed decisions during the drug design and optimization process.

### 4.5 AI in PDC information and database management

The synergy between AI and databases is crucial for extracting insights from extensive datasets and predicting drug-likeness patterns, which are essential for the innovation of PDCs. Through AI-integrated with PDC information and database management, these resources will be instrumental in accelerating the identification and development of novel therapeutic agents.

A recently developed PDCdb (https://pdcdb.idrblab.net/) exemplifies the systematic compilation of biological and pharmaceutical data for a comprehensive list of PDCs. The database includes a wealth of activity data derived from clinical trials, animal models, and cell line studies, amounting to 1,684, 613, and 2,753 data points, respectively (Sun et al., 2025). It provides a comprehensive compilation of ADME properties, plasma half-life, and administration methods for each PDC, as well as an in-depth examination of the chemical modifications, primary targets, modes of action, and conjugation characteristics of the peptide/linker/drug components. The accessibility and scale of PDCdb render it an indispensable resource for AI-driven analysis and pattern recognition, which are critical to drug development.

ConjuPepDB (https://conjupepdb.ttk.hu/), which was first introduced in 2021, complements PDCdb by offering a curated collection of PDCs, detailing over 1,600 conjugates from approximately 230 scientific publications (Balogh et al., 2021). The database excels in providing CAS numbers, biomedical applications, and chemical conjugation classifications, thereby enhancing efficient access and analysis of PDC information. Its web interface, designed with user convenience in mind, supports multifaceted searches, including those based on chemical structure, is particularly beneficial for AI algorithms tasked with identifying conjugates with specific chemical attributes. The structure search capability of ConjuPepDB, facilitated by RDKit, enable precise, sub-structure, and similarity searches, streamlining the process of finding conjugates with targeted chemical scaffolds.

### 4.6 Challenges and limitations of AI in PDC design

Presently, AI applications in PDCs still face many limitations (1) Training data scarcity. PDC-specific datasets (e.g., PDCdb). contain <3,000 entries versus >500,000 for small molecules, risking model overfitting (Sun et al., 2025). (2) Clinical translatability. Only 12% of AI-designed PDCs from 2020-2023 entered Phase I trials, partly due to inadequate *in vivo* prediction tools (Hashemi et al., 2024). (3) Regulatory ambiguity. No AI-developed PDC has received FDA approval as of 2024, contrasting with AI-optimized ADCs like Enhertu^®^ (Nakada et al., 2019). However, AI-enhanced PDC candidates also show promise, for examples, MP-0250, a VEGF/HGF-targeting PDC designed using AlphaFold2-based docking, achieved Phase II efficacy with 34% objective response in NSCLC (NCT04088664), while AI-optimized somatostatin analogs in Lutathera^®^ reduced hepatotoxicity by 22% post-marketing (Morgan et al., 2023).

## 5 Conclusion and future perspectives

The research and development of PDCs has witnessed significant advancements in recent years, with PDCs emerging as a promising class of targeted therapeutic agents. This review highlights the key components of PDCs, including peptides, linkers, and payloads, and discusses the limitations hampering their developments. The integration of AI in PDC design and evaluation represents a paradigm shift, offering innovative solutions to the challenges of peptide selection, linker optimization, payload identification, and predictive modeling for PDCs, which is underscored by the recent Nobel awards.

Future PDC research is likely to be characterized by a deeper understanding of the underlying biology, more precise drug design, and the development of novel therapeutics that harness the full potential of targeted drug delivery. As AI algorithms become more sophisticated, they are expected to enhance the precision of PDC design, leading to improved targeting and therapeutic efficacy. The development of more robust *in vivo* prediction systems tailored to PDCs will be crucial for optimizing their pharmacokinetic and pharmacodynamic properties. Additionally, the expansion of comprehensive PDC databases, coupled with advanced AI-driven analysis, will surely facilitate the discovery of new PDC candidates and accelerate the translation of these agents from bench to bedside.
